# Dynamic Energy Balance: An Integrated Framework for Discussing Diet and Physical Activity in Obesity Prevention—Is it More than Eating Less and Exercising More?

**DOI:** 10.3390/nu9080905

**Published:** 2017-08-19

**Authors:** Melinda M. Manore, D. Enette Larson-Meyer, Anne R. Lindsay, Nobuko Hongu, Linda Houtkooper

**Affiliations:** 1 Nutrition Area, School of Biological and Population Health Sciences, Oregon State University, Corvallis, OR 87331, USA; 2 Department of Family and Consumer Sciences, University of Wyoming, Laramie, WY 82071, USA; enette@uwyo.edu; 3 University of Nevada Cooperative Extension, Las Vegas, NV 89123, USA; alindsay@unr.edu; 4 Department of Nutritional Sciences, University of Arizona, Tucson, AZ 85271, USA; hongu@email.arizona.edu (N.H.); houtkoop@email.arizona.edu (L.H.)

**Keywords:** weight management, exercise, physical activity, energy flux, appetite regulation, dynamic energy balance, diet, obesity prevention, nutrition education

## Abstract

Understanding the dynamic nature of energy balance, and the interrelated and synergistic roles of diet and physical activity (PA) on body weight, will enable nutrition educators to be more effective in implementing obesity prevention education. Although most educators recognize that diet and PA are important for weight management, they may not fully understand their impact on energy flux and how diet alters energy expenditure and energy expenditure alters diet. Many nutrition educators have little training in exercise science; thus, they may not have the knowledge essential to understanding the benefits of PA for health or weight management beyond burning calories. This paper highlights the importance of advancing nutrition educators’ understanding about PA, and its synergistic role with diet, and the value of incorporating a dynamic energy balance approach into obesity-prevention programs. Five key points are highlighted: (1) the concept of dynamic vs. static energy balance; (2) the role of PA in weight management; (3) the role of PA in appetite regulation; (4) the concept of energy flux; and (5) the integration of dynamic energy balance into obesity prevention programs. The rationale for the importance of understanding the physiological relationship between PA and diet for effective obesity prevention programming is also reviewed.

## 1. Introduction

Body composition and weight are the sum of numerous factors that regulate and influence the “intake” and “expenditure” sides of the energy balance equation. Although diet and physical activity (PA) are recognized as key players in energy balance, many nutrition educators may not fully understand how they are physiologically linked and that their impact on body weight is highly interrelated, complementary, and synergistic [1]. The role of diet and PA for weight management and obesity prevention is not as simple as ‘eating less’ or ‘exercising more’. Weight management is no longer a ‘diet vs. PA’ or ‘diet and PA’ issue, but an understanding of the synergy and interrelated nature of these two factors [1,2,3,4,5]. Unfortunately, the public is often confused by what they hear and read in the media about weight management [5,6]. Although there is much research to the contrary [7], it is frequently stated that PA does not promote weight loss since obesity has increased while PA has remained constant in the United States [8]. Still others debate whether obesity is “due to lack of exercise” or ‘low energy flux’ [9] or due to a ‘bad diet’ [8]. Malhotra et al. [8] clearly lays the blame for obesity on the ‘junk food industry’s public relations machinery’ and states that one ‘cannot outrun a bad diet’. These mixed and sometimes incorrect messages are confusing for the public. They also convey the message that only one side of the energy balance equation is important for weight management and obesity prevention. Thus, it is imperative that nutrition educators understand and utilize a dynamic energy balance approach when discussing energy balance and weight issues with the public.

Diet can affect energy balance and health beyond just providing energy. For example, daily energy expenditure is influenced by total energy intake (e.g., kcals or kJ consumed), dietary macronutrient composition (percentage of energy from protein, fat, carbohydrate and alcohol) [5,10], the energy density of the diet (kcals or kJ per g of food) [10,11,12,13], and the timing of food intake [14]. These dietary factors can also alter the thermic effect of food (see Table 1) and the type of substrates stored or used for fuel during PA [5,15,16,17].

Similarly, PA or exercise (see Table 1) affects energy balance beyond simply expending energy. Depending on the type, intensity and duration of PA, the amount energy expended, and the type of fuel used, can vary dramatically (e.g., 30 min of running expends more energy than 30 min of walking). PA that increases muscle mass, such as strength training, can also increase resting metabolic rate (RMR) and total daily energy expenditure. Additionally, research shows that PA alters appetite and appetite-regulating hormones (i.e., inducing appetite suppression or promoting hunger), which could ultimately alter total energy intake [24,25,26,27]. Regular and frequent PA also increases energy flux, which is defined as the rate of energy conversion after absorption from food into body tissues for use in metabolism or its conversion into energy stores (Table 1) [20,28]. A higher level of energy flux improves the body’s ability to match energy intake with expenditure and, thus, can make weight management easier [28,29]. Finally, appropriate PA improves muscle mass and strength [21,30], and can increase or maintain bone mass [31]. Together, these factors improve overall body composition and health, which increases an individual’s ability to maintain an active lifestyle and reduces the risk of obesity and chronic disease [7,32].

This paper reviews key concepts nutrition educators need to know about how PA or exercise impact dynamic energy balance, weight management and obesity prevention, appetite regulation, energy flux, and overall health. It also provides examples of how nutrition educators can practically integrate these concepts into obesity prevention programming or discussions of weight management.

## 2. Dynamic vs. Static Energy Balance

**Figure 1:**
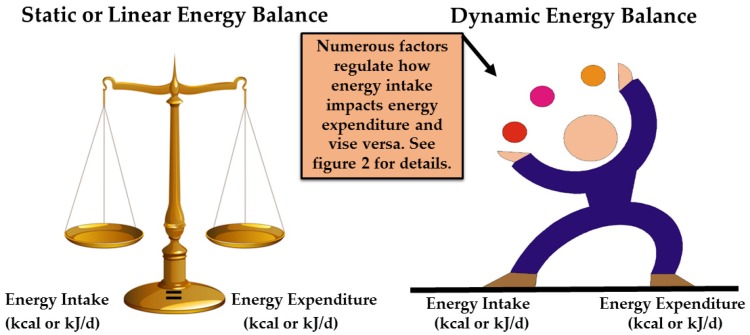
Graphic depiction of the differences between the classic ‘static or linear’ and ‘dynamic’ energy balance models. See Table 1 for definitions and Figure 2 for more details.

Currently, many nutrition and health educators use the classic “static or linear energy balance” approach when discussing weight management or weight loss with the public or their clients (see Table 1 and Figure 1) [3,18]. This approach states that a ‘change in energy stores = energy intake − energy expenditure’ and assumes that by simply changing either side of the energy balance equation weight is gained or lost (e.g., increasing or decreasing 3500 kcal (7700 kJ) will result in a one pound (454 g) weight gain or loss) [18,33]. This approach does not consider individual differences and the numerous factors that change as energy intake or expenditure is altered [34,35,36]. Energy balance is a ‘dynamic’, non-linear process rather than a ‘static’ or linear process [10,35,36]. This means that altering one component of the energy balance equation (i.e., reducing energy intake or increasing energy expenditure) can affect numerous biological and behavioral factors on both sides of the equation in unpredictable and unintended ways [10,35,36,37,38,39].

**Figure 2:**
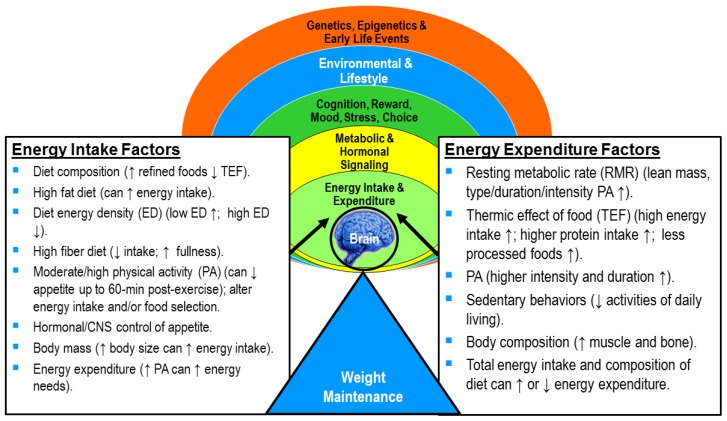
Some examples of the many factors regulating and influencing energy balance. Signals between the brain and body systems sense energy needs and help regulate body weight and composition. Genetics and early life events can affect the body’s ability to sense and manage weight, while environmental and lifestyle factors, including mood, stress, and reward factors, can override body signals for eating. Any change in body size and composition can alter both energy intake and expenditure. Information adapted from Galgani and Ravussin [10], Acheson et al. [11], Manore et al. [17], and Manore [18,19].

Swinburn and Ravussin provide a classic example to illustrate the fallacy of the static energy balance equation under conditions when body weight is changing [40]. Using a 165-pound (75 kg) man they demonstrated how body weight would change if this individual consumed an extra 100 kcal/day (~420 kJ/day) for 40 years [40]. The static energy balance equation would calculate the amount of extra energy consumed to equal ~1.5 million kcals (~6.3 million kJ) with an estimated weight gain of 417 pounds (~190 kg) over the 40-year period. Yet, intuitively nutrition professionals know this probably would not happen. The static or linear energy balance equation does not take into account the increase in energy expenditure that would occur as body weight is gained. As body weight increases, RMR and total energy expenditure also increase due to the greater energy cost of maintaining and moving a larger body. Eventually this individual would achieve energy balance and become stable at a higher body weight. How much body weight is actually gained depends on a number of factors including the energy surplus and macronutrient composition of energy consumed [41], current body composition, type and amount of PA engaged in, and overall energy expenditure. Figure 2 provides examples of the numerous ways diet (energy intake) and PA (energy expenditure) interact to affect our ability to maintain body weight.

To help operationalize the dynamic energy balance model and make it usable for nutrition educators and health professionals, two mathematical models of dynamic energy balance have been developed to better predict body weight changes in response to changes in energy intake and/or energy expenditure over a given time period [38,42]. One model has been developed by Hall et al. [38] at the National Institutes of Health (NIH) [43] and a second model has been developed by Thomas et al. [42] at the Pennington Biomedical Research Center (PBMC) [44]. These models simulate how alterations in energy deficit or excess, which result from adaptations of total energy intake, fuel selection, and energy expenditure, will affect body weight. Both prediction models were developed using weight change results from weight loss studies with overweight or obese adults and thus not be completely applicable to youth, athletes, or individuals who are not overweight. (See Manore [19] for a case study of how these tools can be used with an active individual to predict weight change over a given time period and Webb [45] for how these changes can be incorporated into general weight loss counseling).

## 3. Role of Physical Activity in Body Weight Management

Physical activity can affect body weight in a variety of ways. Below are key ways PA and exercise can help with weight management and prevention of obesity. An understanding of these factors are important when educating clients and the general public about weight loss.

### 3.1. Effect of Physical Activity Quantity on Body Weight and Size

The quantity of PA performed can play an important role in how the body uses the energy consumed from food and the amount of energy flux that occurs (see the next section), and thus further alter the risk for weight gain. Examination of longitudinal data show that when people are more active they are less likely to gain weight over time. For example, Hughes et al. [46] followed weight and body composition changes in older men and women over a nine-year period. Results showed that the men and women who gained weight over this period had significantly lower levels of PA (<480 kcal or 2000 kJ/week) than those who were stable or lost weight (>950 kcal or 4000 kJ/week). In addition, Ekelund et al. [47] followed 288,498 men and women for five years and found that those with higher PA levels has significantly lower waist circumferences, weight gain over time, and a 7%–10% lower chance of becoming obese than those with lower PA. Finally, Shook et al. [48] measured the PA level of young, healthy male and female adults (Body Mass Index (BMI) range = 20–35 kg/m^2^) at baseline and again after 12 months to examine the impact of PA on changes in body weight and fat. Participants were divided into five groups based on PA level. Both body weight and BMI were significantly different across all groups, with the lowest PA level group (~16 min/day moderate/vigorous PA (MVPA); mean = 6062 steps/day) having the highest body weight and BMI. They also found that the differences in body weight were entirely due to differences in fat mass, with the low-PA group having the highest fat mass (30.9 kg or 68 pounds) vs. the highest-PA group (14.2 kg or 32 pounds) (175 min/day MVPA; mean = 10,260 steps/day). See Table 1 for the definition of MVPA. Those in the moderate-PA group (63 min/day MVPA; mean = 7112 steps/day) weighed 13 kg (28.6 pounds) less, while the BMI was 4.4 kg/m^2^ less than the lowest-PA group. After one year, researchers found that the two lowest PA groups had a 1.82 to 3.80 times greater risk of gaining >3% body fat than those participating in the middle or higher PA groups. Thus, low levels of PA are a risk factor for weight gain. The authors concluded that the level of PA associated with the prevention of body weight gain are moderate and achievable.

The research by Shook et al. [48] mentioned above is supported by cross-sectional research examining the relationship between PA and body size using National Health and Nutrition Examination Survey (NHANES) data. These data show that PA level is inversely related to body size in both adults and children. Pate et al. [7] also found that adults participating in MVPA had significantly lower BMI and waist circumference, regardless of age or sex. More recently, Tudor-Locke et al. [49] analyzed adult NHANES data and found that higher step counts were inversely associated with BMI, waist circumference, total body weight, and plasma insulin levels in both men and women. NHANES data on adolescents also reports similar findings. Both Chung et al. [50] and Carson et al. [51] report that body weight status in adolescent boys and girls is inversely related to PA. Carson et al. [52] found that for adolescents (12–19 y) each additional hour per day of MVPA was associated with decreases in waist circumference (~4 cm or 1.6 inches), systolic blood pressure (~4 mmHg) and improved insulin sensitivity (~16%).

### 3.2. Effect of Health-Related Fitness Level on Metabolic Rate

Regular aerobic and resistance exercise can increase health-related fitness levels and muscle mass. With increased health-related fitness, one can work harder and/or longer at the same perceived effort and increase overall energy expenditure. The amount of PA needed to change fitness level depends on the overall energy cost of PA and the type, frequency, duration and intensity of the activity relative to body weight. For example, Shook et al. [53] classified young, healthy adults (*n* = 423; 21–35 years of age; BMI = 25.6 ± 3.8 kg/m^2^) into three levels of fitness (low, moderate, and high) using a treadmill test. They found that after adjusting for body size, those with moderate/high levels of health-related fitness had significantly higher RMR (mL/kg/min) (10%–17% higher) compared to those with low fitness levels. Those in the moderate/high fitness groups also had significantly better fat utilization as assessed by a lower respiratory quotient. Thus, as health-related fitness increases the body becomes better at utilizing fat as a fuel compared to a sedentary individual at the same intensity of PA or exercise [17,54].

### 3.3. Effect of Physical Activity on Muscle and Bone Mass

Muscle and bone mass are key components of body composition; thus, being and staying physically active helps build and maintain muscle and bone mass. Any change in muscle mass can directly affect metabolic rate, substrate unitization, weight management, and overall health [55]. If muscle mass is lost, strength and RMR will decrease, resulting in an overall reduction in total energy expenditure. Muscle mass is gained or maintained through PA and helps prevent the typical decline in RMR seen during periods of energy restriction (e.g., dieting) because muscle mass is more metabolically active than fat mass [56]. Fortunately, the benefits of increased muscle mass can occur without changes in total body weight [57]. As one ages, there are naturally occurring reductions in muscle mass (sarcopenia) and other tissues that contribute to decreasing RMR and increasing risk for gaining body weight [58]. If PA is high enough to cause an increase in muscle mass, bone mass can also increase or be maintained [31].

Weight-bearing PA (see Table 1) helps muscles become and stay stronger and helps form new bone and increase bone strength. Bones become stronger when muscles pull and push against them during PA. Weight-bearing PA is the best type of movement for bones and requires people to work against gravity by having their legs support body weight or by resistance from an external weight [59]. Similar to muscle mass, bone mass declines with age. Bone mass peaks for most people during the third decade of life, after that time bone loss occurs. The rapid phase of bone loss in women starts in menopause and lasts 4–8 years. Bone loss in men typically occurs at a very slow continuous process [22].

Finally, muscle and bone mass are two factors that can improve one’s overall functional ability, which helps maintain PA as one ages. PA facilitates maintaining muscle strength, coordination, and balance; thus, it helps prevent falls and related fractures that are particularly important for older adults and people who have been diagnosed with osteoporosis [59].

### 3.4. Effect of Physical Activity and Energy Restriction on Metabolic Rate

Regardless of the lifestyle modifications used to promote body weight loss, the body readily adapts to the sources of fuel available and degree of energy deficit. When energy restriction alone is used to produce weight loss, approximately 25% of the weight loss is muscle tissue [60]; whereas, when PA is combined with moderate energy restriction, less muscle mass is lost [61]. When body weight is lost due to energy restriction alone, RMR declines, causing total daily energy expenditure to drop below predicted levels based on weight loss alone (e.g., a smaller body needs less energy). However, if PA and moderate energy restriction are combined to achieve weight loss, total daily energy needs are maintained at or above estimated levels [56]. Maintaining higher daily energy needs can make it easier to maintain body weight after ‘the period of energy restriction’ is over. For example, Redman et al. [56] compared two six-month weight loss programs (diet + exercise; diet only) each producing energy deficits of 25%. After adjusting for body weight loss, they found that the diet + exercise program increased total daily energy expenditure by ~200 kcal/day (~837 kJ) vs. the diet-only group, which had a significant decrease in energy expenditure of ~200 kcal/day (~837 kJ). Thus, it could be easier for the diet + exercise group to maintain their reduced body weight because they can eat 200 more kcals/day (837 kJ) than the diet-only group.

### 3.5. Effect of Weight Loss or Gain on Energy Expenditure

Body weight gained or lost can also affect energy expenditure. When body weight is gained, the bigger body requires more energy because there is more body tissue to maintain (e.g., RMR increases), but the body is also less energy efficient because movement can be difficult and requires more energy. Conversely, body weight loss decreases body size and, thus, decreases energy expenditure requirements, yet a smaller person could find it easier to move and; thus, participate in more PA, which can increase energy expenditure. Rosenbaum et al. [62] demonstrated the effect of weight gain or loss on energy needs. They found that when normal body weight participants lost 10% of their body weight, work efficiency increased by 27% (e.g., the same task took less energy), while a 10% weight gain decreased work efficiency by 18% (e.g., the same tasks required more energy). The changes in muscle efficiency (e.g., energy cost), at the altered body weight, accounted for 35% of the change in daily energy expended in PA. Thus, as body weight is gained or lost, the body resists these changes by altering energy expenditure through changes in metabolic rate and work efficiency. These adjustments in energy expenditure could contribute to weight regain after the period of energy restriction is over.

## 4. Role of Physical Activity in Appetite Regulation

Physical activity can also alter appetite, which has the potential to alter total energy intake and, thus, body weight. The effect of PA on appetite and the desire to eat are influenced by the type and intensity of the PA, the environmental temperature, and the characteristics of the exerciser. Thus, the ability of PA to create negative energy balance relies not only on its direct ability to increase energy expenditure but also indirectly on its potential to modulate appetite and/or energy intake. Increases in energy intake that match or exceed the energy cost of an exercise bout (or increased PA) negate body weight loss and can even result in weight gain. A recent review of studies evaluating exercise and weight loss found that “dieters” frequently lost only a third as much weight as was expected, given their energy expenditure during workouts [63]. This phenomenon is more common in women, and is partially explained by compensatory behaviors—including increases in energy intake—that counter exercise energy expenditure and negate body weight loss [63,64]. Compensatory behavior is driven by increased hunger due to exercise or an increased desire to eat (see Table 1). Thus, the energy deficit from a three-mile (~4.8 kilometers) run could easily be reversed by consumption of a 300 kcal (1255 kJ) cookie (or two). Exercise-induced alterations in appetite and their potential effect on energy balance and body weight are summarized below.

### 4.1. Effect of Type and Intensity of Physical Activity on Appetite and Energy Intake

Evolving research suggests that both the type and intensity of PA or exercise influences post-exercise alterations in appetite. Most studies suggest that higher-intensity exercise is more likely to suppress hunger or food intake during the post-exercise period [65,66,67,68,69] than is moderate or light PA. This appetite-suppressing effect seems to last 15–60 min following exercise but can potentially delay the next meal or snack. Lower-intensity PA does not seem to have this same effect. Similarly, the type of PA is important. Research shows that activities such as running, jumping rope, or high-intensity exercise interval workouts suppress appetite [70,71,72], while swimming and walking [71,73,74] are more likely to stimulate appetite and/or food intake. Running seems to have a stronger dampening effect on appetite than does strength training [75]. Overall the types of PA that have the greatest dampening impact on appetite (and favoring negative energy balance) include those that are more intense or which could be considered to jar the gut, such as running.

### 4.2. Effect of Environmental Temperature on Appetite and Energy Intake

The environmental temperature during or following PA can also impact appetite. Cold environments promote hunger and/or food intake whereas hot environments blunt hunger. Recent research has shown that exercising for 45 minutes in cold water (20 °C or 68 °F) promoted an average 44% higher post-exercise (1 h) food intake compared to exercise in neutral (32 °C or 89 °F) conditions [76,77]. Differences in environmental or body core temperature [77,78] could be another reason why swimming seems to promote hunger compared to other types of PA.

### 4.3. Factors that Drive Hunger and Desire to Eat after Exercise

Alterations in key appetite regulating hormones including the hunger hormone ghrelin and the satiety hormones peptide YY (PYY), glucagon-like peptide-1 (GLP-1), and leptin are thought to partially drive appetite changes during and after PA [79]. However, the changes in appetite, and subsequent food intake after PA, are not driven by appetite-regulating hormones alone, since these hormones are influenced by a variety of factors (e.g., exercise intensity, body composition, thirst, sex, energy restriction). In addition, these physiological regulators of appetite can be overridden by eating. For example, after exercising, some individuals can easily “eat back” the energy burned during exercise with an energy-dense snack or calorie-containing beverage, thereby countering the energy cost of the previous bout of PA.

### 4.4. Differences Between Men and Women

Studies consistently suggest that women are more prone to compensatory behaviors, or “eating back” energy expended during exercise by increasing energy intake, thereby negating body weight loss [63,64,80], although such compensatory behaviors do occur in both sexes. It is not known what factors drive this sex difference or whether increased health-related fitness, through regular engagement in PA, dampens compensatory behavior. Recognizing that women and men can unconsciously or consciously engage in compensating energy intake, which can be driven by hunger or food rewards, is important for education about exercise-associated body weight loss.

## 5. Energy Flux: Putting It All Together

After food is digested and absorbed, energy flux refers to the rate of energy conversion for either energy expenditure or transformation to storage (see Table 1) [20]. Thus, energy flux represents the amount of energy moving through the body each day (e.g., higher energy expenditure requires a higher level of energy intake to maintain body weight and body systems). Maintaining a high energy flux (e.g., maintaining a higher level of PA and matching energy intake) could be key to successful weight maintenance, preventing excess weight gain, or maintaining weight loss in the following ways:

- Maintains overall higher energy expenditure by maintaining muscle mass, thermic effect of food, and a higher RMR (e.g., in high energy flux), in addition to the energy expended in PA. A person in high energy flux will expend more energy in PA and need to eat more food to cover their energy needs.
- Heightens sensitivity to appetite control through its impact on appetite-regulatory hormones and food preferences. Thus, the desire to overconsume food is dampened and the total energy intake modified.
- Allows for more appropriate energy intake or volume of food consumed, thus, reducing the probability of overeating. Sedentary individuals (e.g., in low energy flux) can have daily energy needs that are so low that it is easy to consume more food (e.g., kcals) than needed in our current obesogenic environment.

Energy flux plays an important role in helping with weight maintenance, preventing excess weight gain, and maintaining weight loss after “the diet is over” [29]. However, for weight loss to occur either diet, exercise, or both need to be altered in such a way as to sustain a ‘negative energy balance’ or a ‘larger gap between intake and expenditure’ over an extended period of time. Research shows that maintenance of fat free mass (FFM) is better if both diet and exercise are included in a weight loss program compared to diet alone [81]. For most sedentary, overweight, or obese individuals, key minimum changes in diet and exercise need to occur for significant weight loss to be realized. First, research shows that PA needs to increase to at least 250 min/day to achieve clinically significant weight loss [61]. Second, energy intake needs to decrease [81], but not so dramatically as to increase the loss of FFM or suppress metabolic rate [61]. Typical recommendations for diet are to decrease energy intake to a level that produces weight loss, but allows for adequate PA, is above the energy cost of RMR [61], and includes low energy-dense foods and dietary food choices that can be sustained [82].

## 6. Integrating Dynamic Energy Balance into Obesity Prevention Programs

Understanding the integrated roles that both diet and PA play in effective weight management and obesity prevention underpins development of the skills required by nutrition educators to integrate this knowledge into their educational programs. The key points below will be helpful when integrating and applying the importance of dynamic energy balance in educational programing that addresses obesity prevention.

- Diet and PA are both important for effective obesity prevention. **Action**: Incorporate both into obesity prevention programs.
- Energy balance is a dynamic process that is constantly changing depending on numerous diet and PA factors. **Action**: Use Figure 1 and Figure 2 as a teaching tool. Explain the many factors involved in dynamic energy balance and how each can affect body weight.
- Physical activity improves one’s ability to attain and maintain a desirable body weight and body composition (i.e., lower body fat mass). **Action**: Incorporate the key points in section 3.0 to obesity prevention programing. Provide examples to help illustrate how PA works to help manage weight and improve body composition beyond just energy expenditure.
- Physical activity promotes appetite regulation through the body’s appetite-regulatory hormones. **Action**: Explain effects of type and intensity of PA and environmental temperature on appetite and energy intake. Recommend consuming food slowly to allow appetite hormones to positively impact satiety and decrease hunger, consuming foods higher in fiber (whole fruit, vegetables, and grains) to slow eating and increase a sense of fullness. Encourage PA and foods that positively affect appetite and amount of energy intake. Discourage food rewards after exercising because the energy expended can be easily “eaten back”.
- Physical activity promotes higher energy flux, which may make it easier to match energy needs with expenditure. **Action**: Explain how high energy flux is key to successful weight maintenance, preventing excess weight gain, or maintaining weight loss. Demonstrate how to design a personal diet and PA program that helps an individual attain a higher level of energy flux. An effective way to prevent obesity is to maintain a higher level of PA.
- Dynamic energy balance tools can help nutrition educators understand how changes in energy intake and expenditure affect body weight and composition over time. **Action**: Provide examples from these tools to help clients better understand the dynamic energy balance concept.

There are a number of educational programs and resources that incorporate the dynamic energy balance concept, are available to nutrition educators. Use these programs to build diet and PA knowledge, skills, and practices. Then incorporate these new skills in nutrition education programing to help people achieve and maintain a healthy body weight and composition. These resources are listed in Table 2.

## 7. Conclusions

Understanding the dynamics of energy balance and the synergistic and interrelated role that diet and PA play in weight management is important for the development and implementation of effective obesity prevention programs. If obesity prevention efforts only focus on diet and nutrition, or place a limited emphasis on PA, these efforts will likely fail. How an individual’s body weight changes respond to changes made in diet and PA will depend on numerous factors such as health-related fitness level, body composition (i.e., relative muscle mass vs. fat mass), metabolic rate, regulatory hormones, appetite, and the level of energy flux. Understanding dynamic energy balance will help nutrition educators increase their knowledge and comfort level with PA so they can build the skills required to explain how diet and PA work synergistically to help consumers achieve and maintain a healthy body weight and composition. This in turn will help consumers understand that PA does more to influence body weight and composition than just burning calories, and that PA must be done in combination with healthy dietary approaches to achieve effective weight management and obesity prevention.

## Figures and Tables

**Table 1:** Definition of terms frequently used when discussing diet and physical activity (PA) for weight management.

Terms	Definition
Static (linear) energy balance ^a^	Assumes that a change in one side of the energy balance equation (e.g., energy intake) does not change or influence the other side of the equation (e.g., energy expenditure).
Dynamic (non-linear) energy balance ^a^	Assumes that numerous biological and behavior factors regulate and influence both sides of the energy balance equation. Thus, a change in factors on one side of the equation (e.g., energy intake) can and does influence factors on the other side of the equation (e.g., energy expenditure).
Dietary energy density	The energy content of food by weight (kcal or kJ/per gram).
Thermic effect of food	Energy required digesting, metabolizing, or storing energy as fat or glycogen.
Energy flux ^b^	The rate of energy conversion after absorption from food into body tissues for use in metabolism or its conversion into energy stores.
Physical activity ^c^	Bodily movement that enhances health such as walking, dancing, biking, and yoga.
Exercise ^c^	Physical activity that is planned, structured, repetitive, and performed with the goal of improving health or fitness.
Health-related Fitness ^c^	Cardiovascular or muscular fitness focused on the reduction of chronic disease risk.
Moderate-Vigorous PA ^c^	Moderate PA is an intensity of exercise similar to walking at 3.0 miles per hour, while vigorous PA is an intensity of exercise equivalent to running a 10-minute mile.
Weight-bearing PA ^d^	Physical activity such as walking, jogging, running, hiking, dancing, stair climbing, lifting weights, jumping, playing tennis, basketball, or soccer.
Body Composition ^e^	The percentage or amount of fat and fat free (mineral, protein and water) in bone, muscle, and other tissues in the body.
Compensatory Behavior ^f^	Partial or completely compensation, through diet, for the energy expended in exercise (e.g., eating back energy expended during exercise by increasing energy intake), thereby negating body weight loss due to increased PA. Decreasing PA could also be a compensatory behavior.

^a^ Definition from Manore [10,18,19], ^b^ Hand et al. [20], the ^c^ 2008 Physical Activity Guidelines for Americans [21], the ^d^ Surgeon General Report—Bone and Osteoporosis [22], ^e^ Ackerland et al. [23] and ^f^ Stensel [24].

**Table 2:** Diet and physical activity (PA) resources designed for nutrition educators to use in their obesity prevention programs or to recommend to consumers to improve diet and PA.

Program Name/Resource	Web Link
President’s Council on Fitness, Sports and Nutrition	https://www.hhs.gov/fitness/index.html
2008 PA Guidelines for Americans	https://health.gov/paguidelines/
2015 Dietary Guidelines for Americans	https://health.gov/dietaryguidelines/2015/guidelines/
USDA SuperTracker	https://www.supertracker.usda.gov/
USDA SuperTracker My Plate	https://www.choosemyplate.gov/tools-supertracker
Centers for Disease Control & Prevention—Strategies to Prevent Obesity	https://www.cdc.gov/obesity/strategies/index.html
SNAP-Education Toolkit. Obesity Prevention Interventions & Evaluation Framework	https://snapedtoolkit.org/
United Kingdom (UK) Eatwell Guide and Public Health England Government Dietary Recommendations	https://www.gov.uk/government/publications/the-eatwell-guide
UK Obesity Prevention	https://www.nice.org.uk/guidance/cg43
Clinical Practice Guidelines for the Management of Overweight and Obesity Adults, Adolescents and Children in Australia (2013)	https://www.nhmrc.gov.au/guidelines-publications/n57
Canadian Task Force on Prevention Heath Care Guidelines. Obesity in Children	http://canadiantaskforce.ca/guidelines/published-guidelines/obesity-in-children/
Canadian Task Force on Prevention Heath Care Guidelines. Obesity in Adults	http://canadiantaskforce.ca/guidelines/published-guidelines/obesity-in-adults/
World Health Organization. Obesity: Prevention and managing the global epidemic	www.who.int/nutrition/publications/obesity/WHO_TRS_894/en/
